# Coordinated Assembly of the Bacillus anthracis Coat and Exosporium during Bacterial Spore Outer Layer Formation

**DOI:** 10.1128/mBio.01166-18

**Published:** 2018-11-06

**Authors:** Tyler J. Boone, Michael Mallozzi, Alex Nelson, Brian Thompson, Mark Khemmani, Dörte Lehmann, Alexis Dunkle, Paul Hoeprich, Amy Rasley, George Stewart, Adam Driks

**Affiliations:** a Department of Microbiology and Immunology, Loyola University Chicago, Maywood, Illinois, USA; b Department of Veterinary Pathobiology and Bond Life Sciences Center, University of Missouri, Columbia, Missouri, USA; c Biosciences and Biotechnology Division, Lawrence Livermore National Laboratory, Livermore, California, USA; University of Texas Houston Medical School; Harvard University

**Keywords:** *Bacillus anthracis*, assembly, coat, exosporium, spore

## Abstract

Bacterial spores produced by the *Bacillales* are composed of concentric shells, each of which contributes to spore function. Spores from all species possess a cortex and coat, but spores from many species possess additional outer layers. The outermost layer of Bacillus anthracis spores, the exosporium, is separated from the coat by a gap known as the interspace. Exosporium and interspace assembly remains largely mysterious. As a result, we have a poor understanding of the overarching mechanisms driving the assembly of one of the most ubiquitous cell types in nature. To elucidate the mechanisms directing exosporium assembly, we generated strains bearing mutations in candidate exosporium-controlling genes and analyzed the effect on exosporium formation. Biochemical and cell biological analyses argue that CotE directs the assembly of CotO into the spore and that CotO might be located at or close to the interior side of the cap. Taken together with data showing that CotE and CotO interact directly *in vitro*, we propose a model in which CotE and CotO are important components of a protein interaction network that connects the exosporium to the forespore during cap formation and exosporium elongation. Our data also suggest that the cap interferes with coat assembly at one pole of the spore, altering the pattern of coat deposition compared to the model organism Bacillus subtilis. We propose that the difference in coat assembly patterns between these two species is due to an inherent flexibility in coat assembly, which may facilitate the evolution of spore outer layer complexity.

## Abstract (precis)

This work dramatically improves our understanding of the assembly of the outermost layer of the B. anthracis spore, the exosporium, a layer that encases spores from many bacterial species and likely plays important roles in the spore’s interactions with the environment, including host tissues. Nonetheless, the mechanisms directing exosporium assembly into a shell surrounding the spore are still very poorly understood. In this study, we clarify these mechanisms by the identification of a novel protein interaction network that directs assembly to initiate at a specific subcellular location in the developing cell. Our results further suggest that the presence or absence of an exosporium has a major impact on the assembly of other more interior spore layers, thereby potentially explaining long-noted differences in spore assembly between B. anthracis and the model organism B. subtilis.

## INTRODUCTION

**FIG 1:**
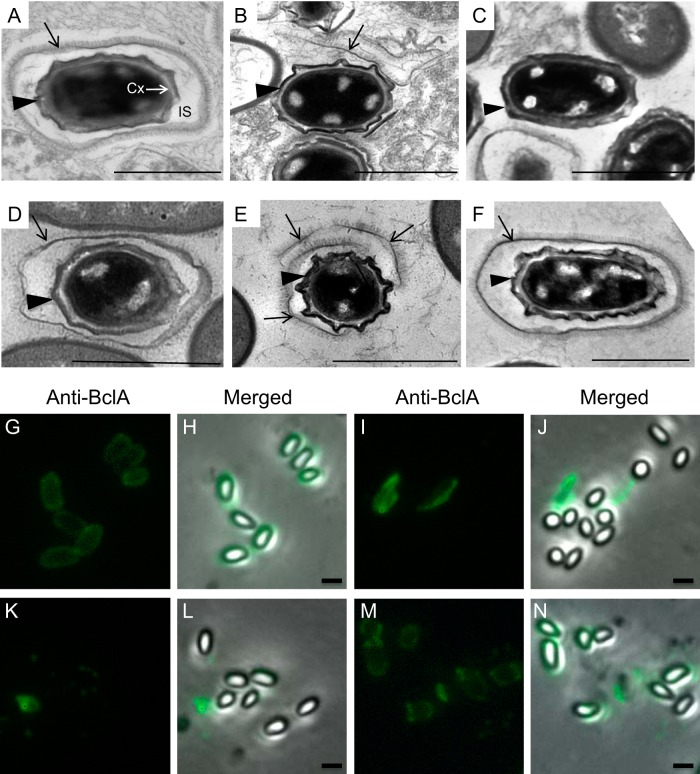
Mutations in *cotO* result in the loss of the exosporium. Wild-type (A, G, and H), MGM76 (*cotO*ΩpMGM3) (B, I, and J), TJB124 (*cotO* mutant) (C, K, and L), TJB130 (*cotO eag1*ΩpTJB10) (D, M, and N), TJB116 (Sterne 34F2 [pMK4-*cotO*]) (E), and TJB239 (Sterne Δ*cotY*) (F) spores were analyzed by thin-section TEM (A to F) and immunofluorescence microscopy (G to N). Arrows indicate the exosporium, while arrowheads indicate the coat. In panel A, the cortex (Cx) and interspace (IS) are also indicated. Images are representative of at least 80 spores visualized. The presence of the exosporium was also assessed by immunofluorescence microscopy and staining with anti-BclA antibodies (G to N). Images are representative of more than 100 spores visualized in three separate spore preparations. Scale bars = 1 μm.

The bacterial endospore, an abundant cell type found in many environments (including the soil and human gastrointestinal [GI] tract), is highly resistant to diverse stresses and survives under extreme conditions for extended time periods. These properties are important for persistence in the environment, colonization of the human host, and pathogenesis (1–3). The spore is organized as a series of layers surrounding a DNA-containing core, each of which makes a critical contribution to resistance and/or dormancy. In the case of Bacillus anthracis, the causative agent of anthrax, these layers are the cortex, coat, interspace, and exosporium (Fig. 1A) (3, 4). The interspace and exosporium are present in a subset of spore-forming species, both pathogenic and nonpathogenic, while the coat and cortex are present in all species (5). Ongoing investigations in B. anthracis and the model organism Bacillus subtilis (which lacks the interspace and exosporium) seek to identify the functions of these layers and elucidate the mechanisms directing their assembly.

Our understanding of interspace and exosporium function remains poor. Exosporium surface properties affect interactions with surfaces and cells in the environment, as well as early interactions with the host upon infection (4, 6–8). The exosporium also affects germination (the process by which spores return from dormancy to active metabolism) and may provide resistance to some stresses (9–11). However, B. anthracis spores lacking the exosporium are largely functional (12) and cause significant disease in animal models (13, 14). These results argue that the exosporium is not required for infection *per se* but may play an important role in natural infection or in other environmental adaptations. Importantly, exosporia are present in many species throughout the *Bacillales* and *Clostridiales* that are not known to cause disease in mammals (15). The exosporium likely has additional unknown functions in B. anthracis, as well as in the large number of other phylogenetically diverse species possessing this structure.

**FIG 2:**
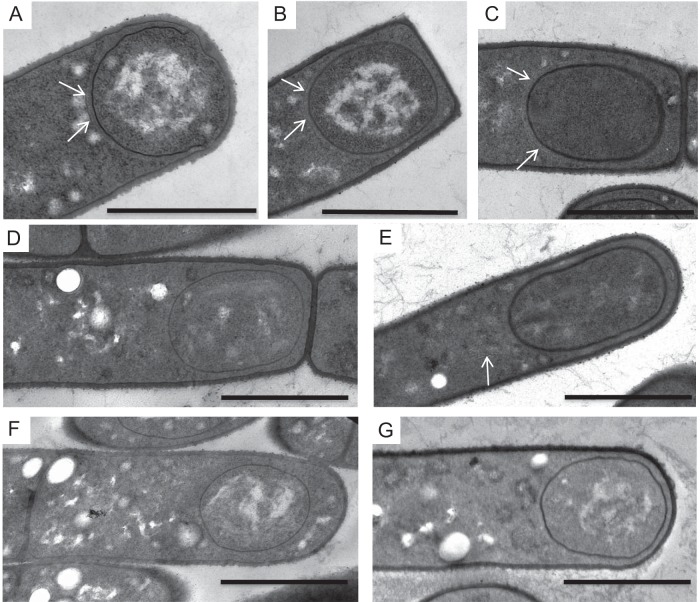
Mutations in *cotO* and *cotY* result in loss of the cap. Wild-type (A to C), TJB124 (*cotO* mutant) (D), MGM76 (*cotO*ΩpMGM3) (E), TJB116 (Sterne 34F2 [pMK4-*cotO*]) (F), and TJB239 (*cotY* mutant) (G) sporangia were harvested at T_2_ (A), T_3_ (B and D to G), and T_4_ (C) and visualized by thin-section TEM. Sporangium preparations were assessed for the presence of a cap, which are indicated by arrows in panels A to C and E. Images are representative of more than 50 sporangia from each strain visualized at these time points. Scale bars = 1 μm.

Elucidating the mechanisms controlling interspace and exosporium assembly is an especially intriguing problem. These mechanisms will almost certainly be novel, as these two layers are morphologically distinct from the better-studied coat. In B. anthracis and the related species B. cereus and B. thuringiensis, the exosporium is composed of a protein shell, the basal layer, which is studded with hair-like projections (Fig. 1A) (10, 12, 16–20). Exosporium assembly initiates soon after the first hallmark event in sporulation, the appearance of the asymmetrically positioned sporulation septum (2 h into sporulation) that divides the cell into a smaller compartment (the forespore) and a larger compartment (the mother cell) (13, 15). The mother cell then engulfs the forespore, resulting in a free protoplast that develops into the spore. Before engulfment is complete, a thin layer of material, the cap, appears at the mother cell side of the septum (Fig. 2A) (13). At these early time points, the cap lacks the electron density and hair-like projections of the mature exosporium, and the gap between it and the forespore (the interspace) is narrower than the interspace seen in the mature spore (13).

The next stage in exosporium assembly is encirclement of the forespore and, ultimately, closure into a contiguous shell. Full encirclement does not occur until six hours into sporulation (12), concomitant with the appearance of the coat and widening of the interspace (13). Encirclement starts at the same time as the initial expression of *exsY*, encoding a major basal layer structural protein (10, 12, 21, 22). The hair-like projections, composed of the proteins BclA, BclB, and possibly others (23–26), are anchored to the basal layer proteins ExsFA/BxpB and ExsFB (19, 27, 28).

The current description of exosporium assembly leaves at least two fundamental mechanistic questions unanswered: (i) what directs the cap to form at the forespore? and (ii) what guides exosporium elongation to form a closed shell around the forespore, rather than disconnected sheets in the mother cell cytoplasm? Addressing these questions is essential to a robust explanatory model of the assembly of this distinctive and unique cell layer, both in B. anthracis and other species. Our incomplete understanding of exosporium assembly impedes the development of improved anthrax therapeutics and spore decontamination strategies that are effective, yet gentle enough for practical needs, because improving these technologies will likely rely on a deeper understanding of exosporium composition (29, 30).

Despite the morphological differences between B. subtilis and B. anthracis spores, previous work shows that several proteins with important roles in spore assembly in these two species are conserved evolutionarily and, at least in some cases, functionally (6, 12, 13). Studies in B. subtilis led to the identification of so-called morphogenetic proteins, which guide assembly of the other coat proteins into their respective layers (31–34). Orthologues of the B. subtilis morphogenetic proteins SpoIVA, CotE, and CotZ have major roles in B. anthracis coat and exosporium formation (10, 12, 13, 21, 31, 35–37).

In this study, we sought to further elucidate the mechanisms directing cap formation and exosporium elongation by characterizing the role of the B. anthracis orthologue of the B. subtilis coat protein gene *cotO.* Our results demonstrate that *cotO* is important for cap formation and assembly of the exosporium, similar to the role of *cotE* (13). We propose a model in which CotE and CotO form a transient network linking the exosporium to the forespore during development. Our results also provide mechanistic insight into a significant difference in coat assembly between B. subtilis and B. anthracis (15, 20, 38), suggesting that the cap alters the pattern of coat assembly by transiently interfering with the deposition of coat proteins. These results emphasize the importance of exploring differences in coat and exosporium assembly among species if we are to have an accurate understanding of the assembly mechanisms for these ubiquitous and environmentally important cell types.

## RESULTS

### *cotO* is required for exosporium formation.

Previously, we demonstrated that inactivation of the B. anthracis *cotO* gene by an insertion-disruption mutation (in strain MGM76), resulted in spores lacking the exosporium (6). Analysis of spore morphology by transmission electron microscopy (TEM) and immunofluorescence microscopy (IFM), using antibodies against the exosporium protein BclA (11), revealed that the mutant spores had an intact coat, but 94% of the spores lacked the exosporium (Fig. 1A, B, and G to J). Exosporium fragments not attached to spores were readily detected. IFM analysis of unwashed spores revealed that 22% had exosporia. These results suggest that exosporium material is synthesized in this strain and may be loosely associated with the spores, but it does not form a closed shell around the spore. As a result, the exosporium is readily sloughed off during either sporulation or washing.

We generated a new mutant strain missing the *cotO* open reading frame (TJB124). The resulting spores also had an intact coat, but ∼96% lacked the exosporium (Fig. 1C). We also observed exosporium fragments disconnected from spores in this strain (Fig. 1K and L); however, these fragments were smaller than those seen in MGM76 spores (Fig. 1I and J), suggesting that exosporium elongation may be reduced in strain TJB124. Despite lacking the exosporium, we did not detect a difference in the patterns of extractible proteins between this strain and the wild type (see Fig. S1 in the supplemental material). There was also no difference in germination (Fig. S2).

10.1128/mBio.01166-18.1 FIG S1 SDS-PAGE of proteins extracted from released spores or sporangia. Washed spores were resuspended in Laemmli buffer, and proteins were extracted by incubating at 100°C for 5 min, followed by 1 min of vortexing. Samples were heated and vortexed 3× and centrifuged at 17,000 × *g* for 5 min, and the supernatant was collected. The concentration of protein extracted was measured using a NanoDrop spectrophotometer (Thermo Fisher Scientific), and 120 µg of protein was separated via 15% (A) or 10% (B) SDS-PAGE gels. Sporangial proteins were extracted by resuspending cell pellets in Laemmli buffer and were mixed with 0.1 mm glass beads. Mother cell lysis was achieved, and forespore proteins were extracted by bead beating, immediately followed by three rounds of incubation at 100°C for 5 min and 1 min of vortexing. Samples were then centrifuged at 17,000 × *g* for 5 min, and the supernatant was collected. The concentration of protein extracted was measured using a NanoDrop spectrophotometer, and 120 µg was separated via 15% SDS-PAGE gels (C). Gels were stained with Bio-Safe Coomassie G-250 stain (Bio-Rad). Download FIG S1, PDF file, 2.2 MB. Copyright © 2018 Boone et al. 2018 Boone et al. This content is distributed under the terms of the Creative Commons Attribution 4.0 International license.

10.1128/mBio.01166-18.2 FIG S2 Effect of the exosporium on germination of *B. anthracis* spores. Wild-type spores or spores lacking an exosporium (*exsY* or *cotO*) were germinated using a combination of inosine and alanine (A), inosine and serine (B), or alanine alone (C). For spores germinated using inosine and alanine, 100 µl of a stock solution containing 50 mM alanine and 10 mM inosine was used to induce germination in a total volume of 1 ml. This stock solution was either used directly or diluted 1:100 or 1:150 before use. Inosine and serine were used at final concentrations of 1 mM or 0.2 mM. Alanine alone was used at final concentrations of 100 mM or 50 mM. Download FIG S2, EPS file, 1.8 MB. Copyright © 2018 Boone et al. 2018 Boone et al. This content is distributed under the terms of the Creative Commons Attribution 4.0 International license.

**FIG 3:**
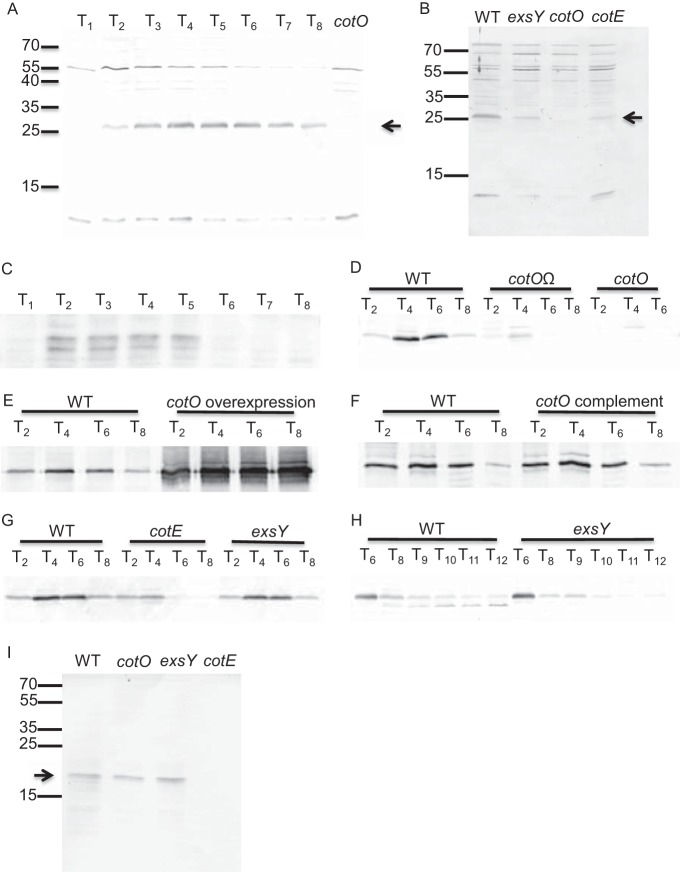
Interaction between CotE, CotO, and ExsY analyzed by Western blotting. Proteins were extracted from wild-type and mutant (strain RG56, *cotE*; strain TJB124, *cotO*; strain MGM76, *cotO*Ω; strain TJB130, *cotO* complement; strain TJB116, *cotO* overexpression; strain TJB139, *exsY*) sporangia or spores. Proteins were extracted from wild-type and mutant released spores (B and I), by three rounds of boiling and vortexing in Laemmli buffer, and mother cell proteins were extracted from sporangia by bead beating in PBS (C). Total sporangial proteins (A, D to H, and J) were extracted by bead beating, followed by three rounds of boiling and vortexing in Laemmli buffer. Forty micrograms of the extracted proteins was separated by 15% SDS-PAGE gels and transferred to a nitrocellulose membrane. Membranes were blocked with 5% BSA and 0.1% Tween 20 in TBS and probed with anti-CotO (1:10,000 in blocking buffer) (A to H) or anti-CotE (1:50,000 in blocking buffer) (I and J) antibodies, followed by anti-rabbit IgG alkaline phosphatase conjugate antibodies (1:10,000 in blocking buffer). Membranes were developed using SigmaFast BCIP/NBT. CotO is first detected in sporangia at T_2_ (A), consistent with the timing of cap formation, and is present throughout sporulation (A and H). When only soluble proteins are extracted from sporangia, CotO is not detectable after T_5_ (C). A band, similar in size to CotO, is present early during MGM76 sporulation (D), suggesting that a fragment of CotO is produced in this strain but may not be stable. CotO is also present early during RG56 sporulation (G) but cannot be detected after T_5_. Less CotO was extracted from the RG56 and TJB139 spores (B), suggesting that its presence in the spore is at least partially dependent on the presence of an exosporium. In comparison, CotE levels were unaffected by the *cotO* and *exsY* mutations (I), consistent with its localization in the coat.

The introduction of *cotO* into the TJB124 genome at an ectopic site (creating strain TJB130) restored the expression of *cotO* (Fig. 3F) and partially restored exosporium formation (Fig. 1D, M, and N). We found that 32% of spores were encircled by an exosporium (Fig. 1D), compared to 4% in TJB124, and an additional 50% of the released spores were partially encircled by an exosporium (Fig. 1M and N). The partially encircling exosporia were not readily sloughed off during washing, unlike the exosporia in strain MGM76 spores, suggesting that these exosporia are more tightly adhered to the underlying spore layer. From these data, we infer that CotO likely has a role in connecting the exosporium to the spore.

Interestingly, no complementation was detected when *cotO* was placed on a nonintegrating plasmid (data not shown). Possibly, this failure was due to increased *cotO* expression. To test the hypothesis that elevated CotO levels cause an exosporium assembly defect, we transformed the wild-type strain with a plasmid expressing *cotO* from its endogenous promoter. The resulting strain (TJB116) had increased CotO levels during sporulation (Fig. 3E), and 88% of the released spores (53 of 60) lacked a contiguous exosporium (Fig. 1E). Most likely, proper exosporium assembly requires CotO concentrations to be within a relatively narrow range.

### *cotO* is required for cap formation.

**FIG 4:**
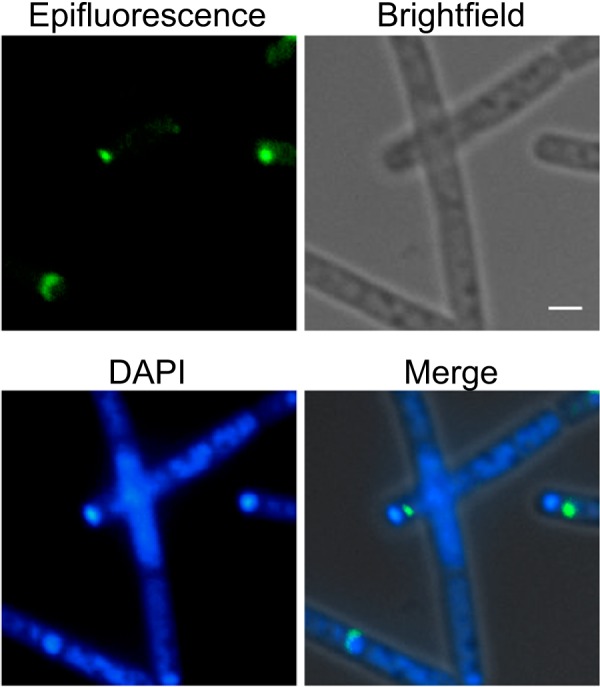
Localization of CotO-GFP to the cap region of the developing spore. Strain MUS8188, bearing a plasmid expressing *cotO*-*gfp*, was harvested at hour two of sporulation and analyzed by epifluorescence and bright-field microscopy. Sporangia were counterstained with 4′,6-diamidino-2-phenylindole dihydrochloride (DAPI) to distinguish the forespore chromosomes (forming tight foci) from the mother cell chromosomes. Scale bar = 1 μm.

To identify the stage at which exosporium assembly requires CotO, we used TEM to analyze sporangia of MGM76 (*cotO*ΩpMGM3) and TJB124 (*cotO*), harvested between 1 and 8 h after the onset of sporulation (T_1_–T_8_). Consistent with previous studies (13, 20), in wild-type sporangia, we first detected the exosporium as a small patch (or cap) of material located at the mother cell-proximal forespore pole during engulfment (Fig. 2A). We also visualized the cap in 54% (42 of 78) of the wild-type sporangia harvested between the completion of engulfment and the appearance of the coat (T_2_–T_5_). At these times, the cap covered less than one-quarter of the two-dimensional circumference of the developing spore and was close (∼35nm) to the forespore membrane (Fig. 2B and C). In contrast, the cap was absent in 93% (65 of 70) of TJB124 (Δ*cotO* mutant) and 80% (45 of 54) of MGM76 (*cotO*ΩpMGM3) sporangia at these times (Fig. 2D and E). Cap-like structures were visible in the mother cell cytoplasm of 6% of TJB124 and 16% of MGM76 sporangia (Fig. 2E) at these times, but they were never observed in wild-type sporangia. These results argue that CotO plays a major role in cap formation and localizing the cap to the forespore. Consistent with a role in cap formation, we first detected CotO in wild-type cells by Western blotting at T_2_ (Fig. 3A), and CotO-green fluorescent protein (CotO-GFP) localized to the mother cell-proximal pole of the forespore at this time (Fig. 4). Our results also suggest that the CotO N-terminus, which is produced in MGM76 (as an almost-full-length protein) (Fig. 3D), retains at least some ability to assemble a cap-like structure.

**FIG 5:**
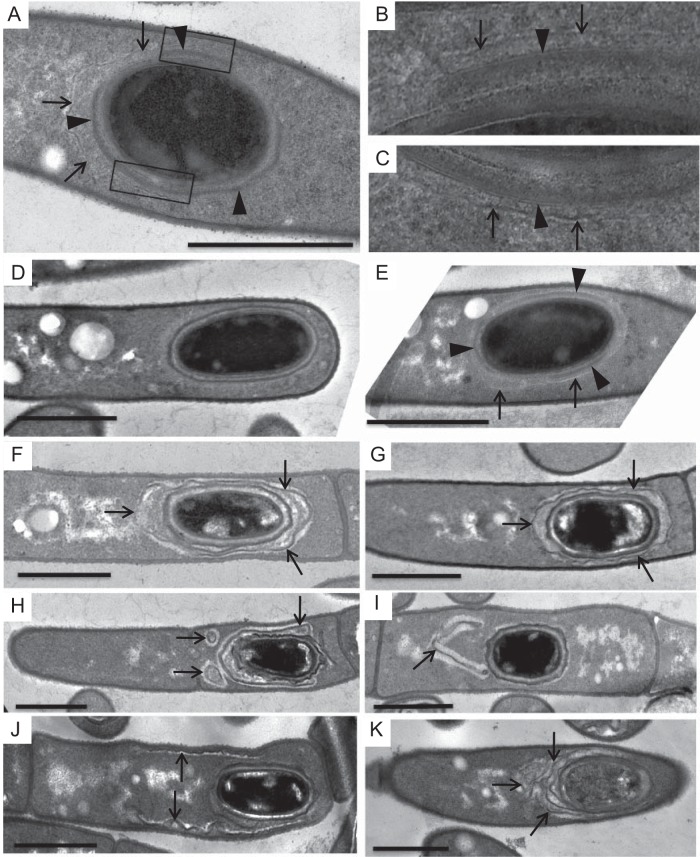
Exosporium formation in wild-type and mutant sporangia. Wild-type (A to C and F), TJB239 (*cotY* mutant) (D, E, and G), TJB124 (*cotO* mutant) (H and I), MGM76 (*cotO*ΩpMGM3) (J), and TJB116 (Sterne 34F2 [pMK4-*cotO*]) (K) sporangia were harvested at intermediate to late stages of sporulation and visualized by thin-section TEM. Sporangia were harvested at T_5_ (A to E), T_6_ (K), T_7_ (H to J), or T_8_ (F and G) to monitor the effects of each mutation on coat and exosporium formation. Arrows indicate the exosporium, while arrowheads indicate densely staining coat material. The areas of panel A enclosed in boxes are magnified in panels B and C to highlight the close association between the leading edge of the elongating exosporium and the coat. At later times, wild-type and TJB239 forespores are completely surrounded by an exosporium (F and G), while forespores from MGM76, TJB124, and TJB116 sporangia were not (H to K). Scale bars = 1 μm.

If CotO is required for cap formation, overproduction of CotO might result in the assembly of additional caps. To address this hypothesis, we used TEM to search for caps in strain TJB116 (Fig. 3E). Prior to T_5_, we did not detect caps (Fig. 2F). Strikingly, we observed multiple (between 6 and 9) short exosporium-like structures in the mother cell cytoplasm of all 21 sporangia visualized at T_6_ (Fig. 5K). We infer that the overexpression of *cotO* resulted in the initiation of multiple cap-like structures, which were too small to visualize by TEM until they began to elongate and gain significant electron density. Possibly, released TJB116 spores lack exosporia because the amount of the major basal layer protein (ExsY) is insufficient to complete multiple exosporia. These data suggest a role for CotO in initiating cap assembly at the mother cell pole.

We expected that CotO would be restricted to the forespore. To test this, we measured CotO steady-state levels in total sporangia and in sporangial fractions containing only soluble mother cell contents (and possibly loosely bound forespore proteins). We detected CotO at all time points (starting at T_2_) in total sporangia (Fig. 3A). However, in the mother cell fraction, CotO was not detectable after T_5_ (Fig. 3C), suggesting that CotO is largely forespore associated after that time.

### *cotY* is required for cap, but not exosporium, assembly.

To better understand the role of the cap in exosporium assembly, we inactivated the *cotY* gene, encoding a major cap protein (10, 12, 21). As expected, the resulting strain (TJB239) lacked a readily detected cap (Fig. 2G). However, consistent with previous observations in B. cereus, virtually all released spores had an exosporium (Fig. 1F) (10).

To assess the effects of the *cotY* mutation on the timing of exosporium formation and the location of exosporium initiation, we compared the degree of exosporium assembly at intermediate times in wild-type and mutant sporangia. We also compared the stage of exosporium assembly to the stage of coat assembly as a separate marker of progression through sporulation. In wild-type sporangia, exosporium elongation occurs simultaneously with coat assembly (Fig. 5A), such that nearly all forespores visualized with a complete coat were also fully surrounded by an exosporium (Fig. 5B). In contrast, in *cotY* mutant sporangia, forespores with a complete coat but no exosporium, or only partially complete exosporium, were readily observed (Fig. 5D and E and S3). In both wild-type and *cotY* mutant sporangia, forespores were fully surrounded by an exosporium by T_6_ (Fig. 5F and G). It was particularly challenging to identify *cotY* mutant sporangia at an intermediate stage of exosporium formation; however, in the 10 sporangia we were able to analyze at this stage, exosporium initiation does not appear limited solely to the mother cell pole of the spore (as in the wild type). Instead, we observed what appear to be small patches of exosporium at random locations around the spore (Fig. 5E and S3). This suggests that exosporium initiation at the mother cell-proximal pole of the wild-type forespore is a result of cap protein deposition prior to or during engulfment. A similar mechanism in B. subtilis directs coat assembly initiation to this same forespore location (15, 38). When exosporium initiation is delayed by the deletion of *cotY*, exosporium assembly can initiate at any location on the forespore surface.

10.1128/mBio.01166-18.3 FIG S3 Effect of early cap formation on the location of exosporium initiation. Sporangia of strain TJB239 (*cotY* mutant), harvested at intermediate time points (T_5_), were visualized by TEM. Sporangia in the early stages of exosporium formation were specifically selected to determine if the timing of exosporium nucleation affects its localization. Arrows indicate the location of small patches of exosporium-like material, which are located at various points around the developing forespore, suggesting that exosporium nucleation is not limited to the mother cell-proximal pole of the forespore in this strain. Download FIG S3, PDF file, 2.5 MB. Copyright © 2018 Boone et al. 2018 Boone et al. This content is distributed under the terms of the Creative Commons Attribution 4.0 International license.

### Possible role for CotO in exosporium elongation around the spore.

Our data suggest that while CotO is required for early cap formation, early cap formation is not essential for exosporium assembly. This suggests that CotO (possibly by interacting with ExsY) also plays a role in directing elongation of the exosporium around the forespore. To investigate the role of CotO in guiding exosporium elongation, we characterized exosporium assembly in the *cotO* mutant strains at intermediate and late times using TEM. In wild-type sporangia, while the interspace widens at the mother cell-proximal forespore pole (Fig. 5A), the leading edge of the encircling exosporium remains very close to the coat (Fig. 5B and C). In contrast, this close association between the coat and exosporium is not seen in *cotO* mutant sporangia. In *cotO* mutant sporangia, the exosporia either partially encircle the forespore (Fig. 5H), are present as dispersed fragments (Fig. 5I), or appear as wide tubes (Fig. 5J). Our data suggest that the exosporium-leading edge is attached to the coat by a protein network, including CotO, and is likely similar or even the same as that connecting the cap to the forespore.

### Cap formation alters the pattern of coat assembly.

**FIG 6:**
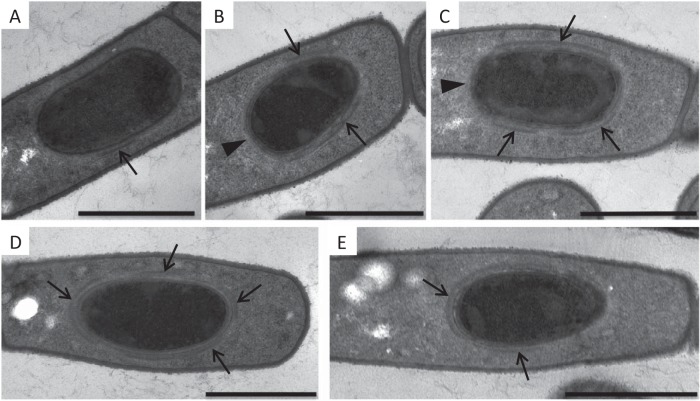
Effect of *cotO* mutations on coat formation. Wild-type (A to C), TJB124 (*cotO* mutant) (D), and MGM76 (*cotO*ΩpMGM3) (E) sporangia were harvested at T_5_ and visualized using thin-section TEM. At this time, many forespores were only partially surrounded by densely staining coat material (arrows), indication that coat formation was actively occurring. A gap in the densely staining coat layer at the mother cell proximal pole of the forespore (arrowheads) was present in nearly all wild-type sporangia (A to C), while densely staining material was often observed at this location in the TJB124 and MGM76 sporangia (D and E). Scale bars = 1 μm.

In B. subtilis, coat assembly begins at the mother cell-proximal pole of the forespore (5, 38). In contrast, coat material first appears on the long axis of the B. anthracis and B. cereus forespore (Fig. 6A to C) (20). This difference in assembly patterns is a strong indication of at least some important difference in coat assembly between B. cereus group and B. subtilis spores. We noticed specifically that in sporangia with partially formed coats, there was a gap in the densely staining coat layer at the mother cell-proximal forespore pole in 80% (41 of 51) of wild-type sporangia (Fig. 6B and C). Ten sporangia lacked gaps at the mother cell pole, but these sporangia were almost completely surrounded by a coat, making it difficult to assess the location of coat initiation. We interpret these data as suggesting that in most sporangia, coat material first accumulates on the long side of the spore instead of the mother cell pole as in B. subtilis.

Our results raised the possibility that the cap, appearing at the mother cell pole prior to coat maturation, interferes with the deposition of some coat proteins at intermediate and late times. If the cap does interfere with coat formation, we hypothesized that sporangia unable to assemble the cap should deposit the electron densely staining coat material first on the mother cell-proximal spore pole. To test this, we used TEM to visualize coat formation in *cotE*, *cotO*, and *cotY* mutant sporangia. Similar to *cotO* and *cotY* mutant sporangia, most *cotE* mutant sporangia fail to form a cap (13). Consistent with our hypothesis, 51% (26 of 51) of *cotO* mutant sporangia, 42% (11 of 26) of *cotE* mutant sporangia, and 35% (9 of 26) of *cotY* mutant sporangia had electron-dense coat material covering the mother cell-proximal pole of the forespore at this stage (Fig. 6D and E, and data not shown), suggesting that the cap may alter assembly of the coat.

### CotE interacts with CotO within a protein network linking the cap to the forespore.

We hypothesized, based on the similarity of the observed *cotO* mutant phenotype with the previously published *cotE* mutant phenotype, that CotE and CotO are part of a protein network linking the exosporium to the developing spore (13). To address this hypothesis, Western blot analysis was used to assess the effect of a *cotE* mutation on CotO steady-state levels or a *cotO* mutation on CotE levels. While a *cotO* mutation had no detectable effect on the amount of CotE extracted from released spores (Fig. 3I), a *cotE* mutation resulted in reduced levels of spore-associated CotO (Fig. 3B). This suggests that CotE has a role in CotO stability, assembly into the spore, or extractability from the spore.

**FIG 7:**
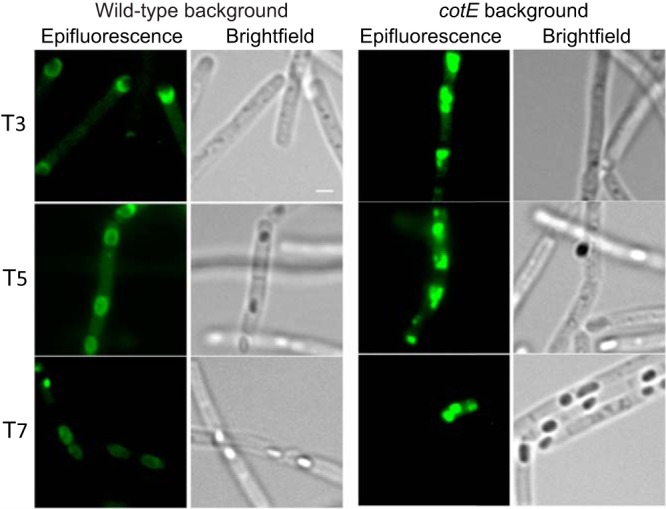
Effect of a *cotE* mutation on the localization of CotO-GFP during sporulation. Strain MUS8188, bearing a plasmid expressing *cotO*-*gfp* (left images), and strain MUS8189, bearing this plasmid and also harboring a *cotE* mutation (right images) were analyzed over the course of sporulation by epifluorescence and bright-field microscopy. Scale bars = 1 μm.

To address the possibility that CotE directs CotO assembly early in sporulation, we used epifluorescence microscopy to monitor the localization of a CotO-GFP fusion protein in wild-type and *cotE* mutant sporangia. We found that in otherwise wild-type sporangia harboring *cotO-gfp* on a plasmid (MUS8188), CotO-GFP localized to, and eventually surrounded, the forespore (Fig. 7). In a strain bearing a mutation in *cotE* (MUS8189), CotO-GFP accumulated in the mother cell cytoplasm early during sporulation but did not localize to the forespore. No fluorescence was visible in this strain at later time points (T_7_). We conclude that CotE has a significant role in CotO localization and may impact CotO stability.

To address the hypothesis that *cotE* is required for CotO stability, we measured CotO steady-state levels throughout sporulation in wild-type and *cotE* mutant sporangia using Western blot analysis. In wild-type sporangia, we detected CotO at all time points after T_2_. In contrast, CotO was not detected after T_5_ in *cotE* mutant sporangia (Fig. 3G). These data, taken together with the observation that CotO-GFP is not detectable at late time points in *cotE* mutant sporangia (Fig. 7), suggest that CotO is likely degraded in the *cotE* mutant spores. Importantly, CotO levels during sporulation were largely unaffected by a mutation in *exsY* (Fig. 3G and H). This suggests that the low level of extractable CotO is due to the loss of CotE specifically and not a result of a mislocalized exosporium.

**FIG 8:**
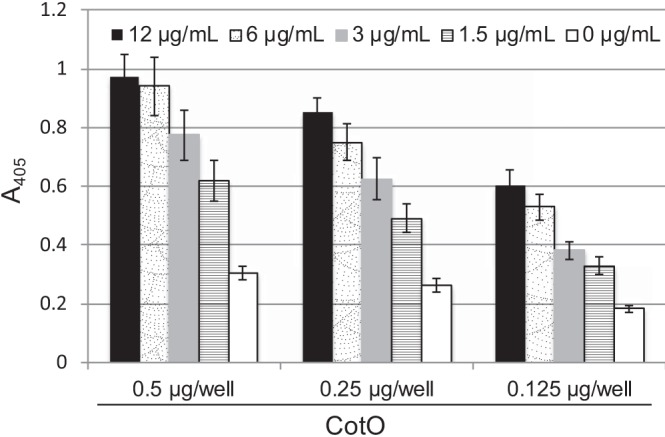
Measurement of the CotO-CotE interaction *in vitro* using ELISA. CotO (0, 0.125, 0.25, and 0.5 µg/well) was fixed to wells of a 96-well plate; the plate was blocked with 5% BSA plus 0.1% Tween 20 and incubated with CotE (0 to 12 µg/ml in blocking buffer). The amount of CotE retained in each well was assessed by ELISA, by application of rabbit anti-CotE (1:5,000) antibodies to the wells, followed by anti-rabbit IgG alkaline phosphatase conjugate (1:2,000) antibodies. Plates were developed using 4-nitrophenol phosphate for 30 min at room temperature before stopping the reaction with NaOH and measuring the absorbance at 405 nm (*A*_405_). Values from wells containing no CotO were subtracted from the values of corresponding wells to account for nonspecific binding.

A simple mechanism by which CotE could recruit CotO to the forespore would be direct interaction between the two proteins. To test whether CotE and CotO can interact directly, we overproduced each protein as a His-tagged fusion protein in Escherichia coli, purified them, and tested their binding using an enzyme-linked immunosorbent assay (ELISA). We found that CotE bound to CotO immobilized to the wells of a 96-well plate in a concentration-dependent manner (Fig. 8). These data indicate that CotE and CotO interact directly *in vitro* and support the hypothesis that CotE and CotO interact during sporulation to direct exosporium assembly.

## DISCUSSION

**FIG 9:**
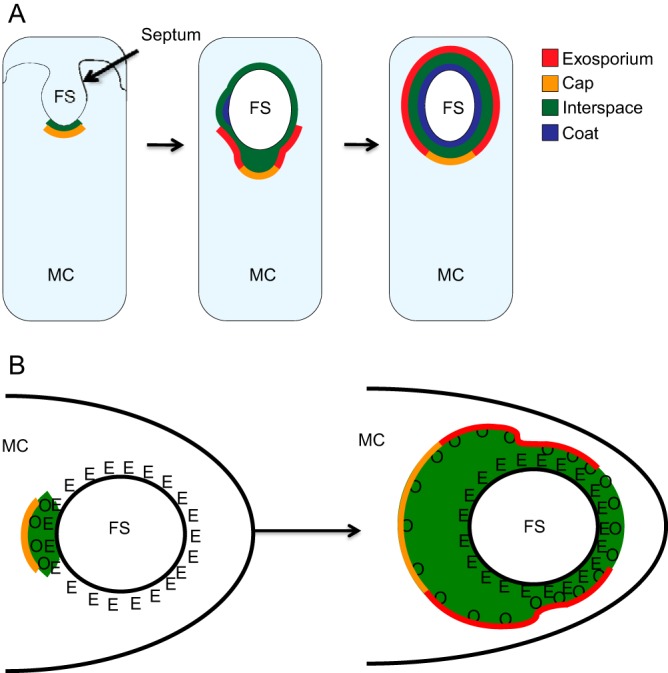
Model of *B. anthracis* spore formation. (A) Model of spore assembly in *B. anthracis*. FS, forespore; MC, mother cell. At an early time in sporulation and before completion of forespore engulfment, a thin layer of material forms on the mother cell side of the septum (green). This layer likely contains at least some coat (SpoIVA, ExsA, CotE, and CotO) and interspace material but is transparent when visualized by TEM. The cap assembles on top of this layer (yellow) (left-most cartoon), resulting in the gap observed by TEM between the cap and the forespore membrane. Soon after engulfment, (i) the thin layer of coat/interspace material assembles around the entire forespore (see Discussion), (ii) exosporium proteins (red) assemble at the cap’s edge, on the interspace material, and proceed to encircle the forespore, while the interspace material underneath the cap region widens, and (iii) densely staining coat features (blue) first appear at a location immediately ahead of the leading edge of the exosporium (middle cartoon). As exosporium assembly continues, the interspace widens along the entire spore, and coat and exosporium assembly complete (right-most cartoon). (B) Roles of CotE (E) and CotO (O) in linking the cap and forespore. CotE (anchored to the surface of the coat, which contains SpoIVA and ExsA) and CotO (at the underside of the cap, yellow) are part of a network which resides in the same location as the interspace (green) and connects the cap and forespore (left-most cartoon). As exosporium assembly proceeds, the interspace widens, and the interactions between CotE and CotO are disrupted throughout most of the interspace. However, at the leading edge of the assembling exosporium, the interspace is thin, and we speculate that the CotE-CotO interaction remains intact until the exosporium elongates completely around the forespore.

In this study, we address a fundamental question in spore formation: how is exosporium formation directed to initiate at one pole of the forespore surface? Our findings argue that, to a large degree, this is achieved by a protein network, consisting at a minimum of the proteins CotE, CotO, and CotY, which assemble at the forespore prior to engulfment (Fig. 9A, left image, and B). The resulting structure, the cap, ensures that continued exosporium assembly proceeds relatively rapidly from a single location (Fig. 9A, middle image). We infer, based on previous work, that the coat proteins SpoIVA and ExsA (the homologue of SafA in B. subtilis) likely reside between CotE and the forespore membrane and anchor the network to the forespore (13, 39–42). This model of assembly resembles the coat protein network in B. subtilis, suggesting that there may be a core outer layer assembly program that is conserved among all spore-forming *Bacillaceae*. Our results also argue that the impact of the cap on coat assembly potentially explains a significant difference in spore morphogenesis between B. anthracis and B. subtilis, the location at which electron-dense coat material first appears. These findings emphasize the limitations of the current model of spore assembly, which fails to account for important features of B. anthracis coat assembly. While coat formation in B. subtilis is well characterized and is the basis for understanding coat formation in all spore-forming species, the observation that the exosporium alters the pattern of coat assembly in B. anthracis argues for a need to thoroughly investigate spore formation in other species with other morphologically diverse outer layers.

The protein network connecting the exosporium to the forespore cannot be static throughout sporulation, since the exosporium migrates away from the forespore during sporulation (Fig. 2A and 5A). We observed that the distance between the cap and forespore first expands from 35 nm at T_4_ to 71 nm with the appearance of densely staining coat material at T_5_. Despite this expansion, the exosporium appears to be tightly associated with the coat at the leading edge of elongation (Fig. 5B and C). Possibly, transient protein-protein interactions maintain the connection between the coat and exosporium, even as new coat proteins are added. We speculate that the network connecting SpoIVA, ExsA, and CotE has a degree of flexibility that accommodates assembly of new proteins into the coat. Following this expansion, the protein network appears to be disrupted, as a significant portion of the interspace widens during exosporium encirclement. This widening implies that the protein network is further expanded, or disrupted completely, to allow the incorporation of interspace material. We prefer a model in which the network is disrupted, since the gap between the coat and exosporium is not uniform around the spore. We speculate that the disruption occurs between CotE and CotO, with CotE remaining in the coat (13), while CotO either becomes an exosporium protein or is lost from the spore (Fig. 9). A key open question is the contents of the interspace, a problem we are presently investigating.

Our second key finding is that, unlike B. subtilis, not every B. anthracis coat layer initiates at the mother cell-proximal spore pole. Instead, as previously demonstrated in B. cereus, at least some coat layers initiate on the long side of the spore (20). We found that mutations preventing cap assembly result in a pattern of coat assembly resembling B. subtilis. We speculate that assembly of the cap, after the deposition of early synthesized coat proteins (including SpoIVA, ExsA, CotE, and CotO) at the mother cell-proximal pole, occludes further deposition of coat proteins at this location. We argue that coat proteins are unable to diffuse through the cap and therefore must traffic between the exosporium and the forespore membrane surface, resulting in late coat formation proceeding from the edge of the cap. After interspace widening, however, the cap no longer interferes with coat assembly, and electron-dense layers form where the cap used to be. We suggest that the mechanism of coat assembly may be more flexible than previously thought, changing as a result of the evolution of additional outer structures. In this view, no single model of sporulation is likely to completely describe spore formation in all species.

The present work reinforces the view that orthologues of morphogenetic coat protein genes can have very different functions in different species (12, 13, 31, 37). The morphogenetic coat proteins, identified in B. subtilis as being important for proper coat assembly, are often conserved in other *Bacillaceae* (43). Our results add to the growing list of morphogenetic proteins that are important for spore assembly in both B. subtilis and B. anthracis, hinting at a core assembly program that is conserved among *Bacillaceae* (10, 12, 13, 32, 37, 39). Our results also demonstrate that while the sequences of these proteins are conserved, their exact function may not be; like CotE, CotO is important for coat assembly in B. subtilis and exosporium assembly in B. anthracis (13, 31, 32). These results suggest that orthologues of known morphogenetic proteins may play roles in the assembly of other novel outer layers, such as the canoe-shaped parasporal body (CSPB) of Brevibacillus laterosporus or the spikes projecting from the coat of Bacillus clausii (15). We speculate that while unidentified, and species-specific, morphogenetic proteins likely exist (5), it would be very informative to understand how the functions of known morphogenetic proteins diverge in different species. This information would likely reveal currently undetected roles for these proteins and provide insight into the ecological forces driving the diversification of spore outer layers.

## MATERIALS AND METHODS

### General techniques and plasmid construction.

All strains were cultured in Luria-Bertani (LB) medium. When needed, E. coli strains were grown in the presence of spectinomycin (100 μg/ml), kanamycin (25 μg/ml), or carbenicillin (80 μg/ml). B. anthracis strains were grown in the presence of polymyxin B (60 units/ml), kanamycin (20 μg/ml), erythromycin (10 μg/ml), chloramphenicol (10 μg/ml), or spectinomycin (250 μg/ml). Recombinant DNA techniques were performed as previously described (44). PCR products were purified using the QIAquick PCR purification kit (Qiagen). Plasmids (Table S1) were purified using the Wizard plasmid purification kit (Promega). Enzymes used for cloning were purchased from Thermo Fisher Scientific and used according to the manufacturer’s instructions.

10.1128/mBio.01166-18.4 TABLE S1 Primers, plasmids, and strains. Download Table S1, DOCX file, 0.02 MB. Copyright © 2018 Boone et al. 2018 Boone et al. This content is distributed under the terms of the Creative Commons Attribution 4.0 International license.

### Generation of mutant B. anthracis strains.

The *cotO* gene was initially disrupted via Campbell-type single-reciprocal recombination, as previously described (13, 45), resulting in strain MGM76. Markerless deletion mutations were generated in *cotO*, *exsY*, and *cotY*, resulting in strains TJB124, TJB139, and TJB239, respectively, and confirmed by PCR (46, 47). Single-copy complementation of the *cotO* deletion was achieved by integrating a plasmid expressing *cotO* (pTJB126) under its native promoter, immediately downstream of the *eag1* gene (18). To generate a strain of B. anthracis overproducing CotO, a plasmid expressing *cotO* from its native promoter was introduced into B. anthracis by electroporation, as previously described (13, 48, 49).

To generate strains of B. anthracis producing a CotO-GFP fusion protein, *cotO* was inserted into plasmid pDG4099 (pMK4 bearing the enhanced GFP [eGFP] reporter cassette). The resulting plasmid (pBT4653) was introduced into B. anthracis using electroporation, followed by selection with chloramphenicol.

### Sporulation.

B. anthracis spores were generated by growth to exhaustion in Difco sporulation medium (DSM) (13). Spores were then washed three times and resuspended in distilled water. After resuspension in DSM, growth was monitored by measuring the optical density at 600 nm (OD_600_) to determine the initiation of sporulation (T_0_), defined as the end of exponential-phase growth. Two-milliliter samples were taken every hour during sporulation (T_1_–T_12_) and either immediately fixed for analysis by transmission electron microscopy (TEM) (13) or stored at −80°C until proteins could be extracted for Western blot analysis.

### Purification of CotO and CotE and the generation of anti-CotO antibodies.

The CotE and CotO proteins were overproduced and nickel-affinity purified, using the QIAexpressionist system, according to the manufacturer’s instructions (Qiagen). Purified CotO was used to generate anti-CotO antibodies in New Zealand White rabbits (GenScript, Piscataway, NJ), which were protein A affinity purified.

### Protein isolation from spores and sporangia.

Coat and exosporium proteins were isolated from harvested spores by resuspending in Laemmli buffer, followed by three rounds of heating (100°C for 5 min) and then vortexing (1 min) (13). Soluble proteins were extracted from sporangia by resuspending the cells in phosphate-buffered saline (PBS) and bead beating (50). To characterize forespore-associated proteins in sporangia, cells were resuspended in Laemmli buffer and lysed by bead beating, and the forespore-associated proteins were extracted by three rounds of heating (100°C for 5 min) and vortexing (1 min). Samples were then centrifuged at 17,000 × *g* for 5 min, and the supernatant was collected.

### SDS-PAGE and Western blot analyses.

Proteins were quantified using a NanoDrop spectrophotometer (Thermo Scientific), 120 μg was separated via 10% or 15% SDS-PAGE gel, and the gel was stained with Bio-Safe Coomassie G-250 stain (Bio-Rad) (Fig. S1).

For Western blot analysis, 40 μg of extracted proteins was separated by 15% SDS-PAGE gels and transferred to a nitrocellulose membrane (Bio-Rad). Membranes were washed with Tris-buffered saline (TBS; 0.01 M Tris, 0.15 M NaCl [pH 7.5]) and incubated for 30 min with blocking buffer (5% bovine serum albumin [BSA], 0.1% Tween 20 in TBS). Membranes were washed with TBS and incubated with anti-CotE (1:50,000 in blocking buffer [13]) or anti-CotO (1:3,000 in blocking buffer) antibodies overnight at 4°C. Membranes were washed 3 times with 0.1% Tween 20 in TBS (TBST) and 2 times with TBS and incubated with anti-rabbit alkaline phosphatase conjugate antibodies (1:10,000 in blocking buffer) for 1 h. Membranes were washed 3 times with TBST and 2 times with TBS and incubated with SigmaFast 5-bromo-4-chloro-3-indolylphosphate/nitroblue tetrazolium (BCIP/NBT; Sigma-Aldrich) for 5 to 45 min before stopping the reaction with 3 changes of distilled water.

### Immunofluorescence microscopy.

Washed spores (10 µl) were adhered to the wells of a multiwell microscope slide (MP Biomedicals) coated with poly-l-lysine (Sigma-Aldrich). Slides were washed with PBS, treated with blocking solution (2% BSA in PBS) for 30 min, washed 9× with PBS, and incubated for 1 h with either polyclonal anti-BclA (diluted 1:1,000 in blocking buffer; BEI Resources) or anti-CotE (diluted 1:50,000 in blocking buffer) antibodies. Slides were washed 9× with PBS and incubated for 1 h with goat anti-rabbit antibodies conjugated to Alexa Fluor 488 (1:500 in blocking buffer; Molecular Probes). Slides were washed 9× with PBS, and coverslips were mounted using PermaFluor Mountant mounting medium (Thermo Scientific). Spores were then visualized using a Leica DM IRB fluorescence microscope. Images were collected with a MagnaFire cryo-cooled charge-coupled-device (CCD) camera and processed using the ImageJ software.

### *In vitro* interaction between CotE and CotO measured by ELISA.

The potential for CotE and CotO to interact *in vitro* was measured using an enzyme-linked immunosorbent assay (ELISA), as previously described (51). CotO was fixed to 96-well MaxiSorp plates (Thermo Scientific) at concentrations of 0, 0.125, 0.25, and 0.5mg/well and incubated for 1 h with blocking buffer (5% BSA, 0.1% Tween 20 in TBS), before incubating for 1 h with CotE (0 to 12 µg/ml in blocking buffer). The amount of CotE retained in the wells was determined using anti-CotE antibodies (1:10,000 in blocking buffer), followed by anti-rabbit-IgG alkaline phosphatase conjugate antibodies (1:2,000 in blocking buffer). Finally, wells were developed for 30 min with 200 µl of 4-nitrophenol phosphate before the reaction was stopped with 50 µl of 3 M NaOH, and the absorbance at 405 nm (*A*_405_) was measured.

## References

[1] Browne HP, Forster SC, Anonye BO, Kumar N, Neville BA, Stares MD, Goulding D, Lawley TD 2016 Culturing of ‘unculturable’ human microbiota reveals novel taxa and extensive sporulation. Nature 533:543–546. doi:10.1038/nature17645. 27144353 PMC4890681

[2] Swick MC, Koehler TM, Driks A 2016 Surviving between hosts: sporulation and transmission, p 368–373. *In* Kudva I, Cornick N, Plummer P, Zhang Q, Nicholson T, Bannantine J, Bellaire B (ed), Virulence mechanisms of bacterial pathogens, 5th ed ASM Press, Washington, DC.

[3] Driks A 2009 The *Bacillus anthracis* spore. Mol Aspects Med 30:368–373. doi:10.1016/j.mam.2009.08.001. 19683018

[4] Stewart GC 2015 The exosporium layer of bacterial spores: a connection to the environment and the infected host. Microbiol Mol Biol Rev 79:437–457. doi:10.1128/MMBR.00050-15. 26512126 PMC4651027

[5] Driks A, Eichenberger P 2016 The spore coat, p 179–200. *In* Driks A, Eichenberger P (ed), The bacterial spore: from molecules to systems. ASM Press, Washington, DC.

[6] Chen G, Driks A, Tawfiq K, Mallozzi M, Patil S 2010 *Bacillus anthracis* and *Bacillus subtilis* spore surface properties and transport. Colloids Surf B Biointerfaces 76:512–518. doi:10.1016/j.colsurfb.2009.12.012. 20074921

[7] Wang Y, Jenkins SA, Gu C, Shree A, Martinez-Moczygemba M, Herold J, Botto M, Wetsel RA, Xu Y 2016 *Bacillus anthracis* spore surface protein BclA mediates complement factor H binding to spores and promotes spore persistence. PLoS Pathog 12:e1005678. doi:10.1371/journal.ppat.1005678. 27304426 PMC4909234

[8] Bozue J, Moody KL, Cote CK, Stiles BG, Friedlander AM, Welkos SL, Hale ML 2007 *Bacillus anthracis* spores of the *bclA* mutant exhibit increased adherence to epithelial cells, fibroblasts, and endothelial cells but not to macrophages. Infect Immun 75:4498–4505. doi:10.1128/IAI.00434-07. 17606596 PMC1951178

[9] Chesnokova ON, McPherson SA, Steichen CT, Turnbough CL Jr. 2009 The spore-specific alanine racemase of *Bacillus anthracis* and its role in suppressing germination during spore development. J Bacteriol 191:1303–1310. doi:10.1128/JB.01098-08. 19074397 PMC2632011

[10] Johnson MJ, Todd SJ, Ball DA, Shepherd AM, Sylvestre P, Moir A 2006 ExsY and CotY are required for the correct assembly of the exosporium and spore coat of *Bacillus cereus*. J Bacteriol 188:7905–7913. doi:10.1128/JB.00997-06. 16980471 PMC1636315

[11] Severson KM, Mallozzi M, Bozue J, Welkos SL, Cote CK, Knight KL, Driks A 2009 Roles of the *Bacillus anthracis* spore protein ExsK in exosporium maturation and germination. J Bacteriol 191:7587–7596. doi:10.1128/JB.01110-09. 19837802 PMC2786611

[12] Boydston J, Yue L, Kearney J, Turnbough C 2006 The ExsY protein is required for complete formation of the exosporium of *Bacillus anthracis*. J Bacteriol 188:7440–7448. doi:10.1128/JB.00639-06. 16936017 PMC1636282

[13] Giorno R, Bozue J, Cote C, Wenzel T, Moody KS, Mallozzi M, Ryan M, Wang R, Zielke R, Maddock JR, Friedlander A, Welkos S, Driks A 2007 Morphogenesis of the *Bacillus anthracis* spore. J Bacteriol 189:691–705. doi:10.1128/JB.00921-06. 17114257 PMC1797280

[14] Bozue J, Cote CK, Moody KL, Welkos SL 2007 Fully virulent *Bacillus anthracis* does not require the immunodominant protein BclA for pathogenesis. Infect Immun 75:508–511. doi:10.1128/IAI.01202-06. 17074844 PMC1828395

[15] Driks A, Eichenberger P (ed). 2016 The bacterial spore: from molecules to systems. ASM Press, Washington, DC.

[16] Hachisuka Y, Kojima K, Sato T 1966 Fine filaments on the outside of the exosporium of *Bacillus anthracis* spores. J Bacteriol 91:2382–2384. 4957618 10.1128/jb.91.6.2382-2384.1966 PMC316223

[17] Sylvestre P, Couture-Tosi E, Mock M 2002 A collagen-like surface glycoprotein is a structural component of the *Bacillus anthracis* exosporium. Mol Microbiol 45:169–178. doi:10.1046/j.1365-2958.2000.03000.x. 12100557

[18] Sylvestre P, Couture-Tosi E, Mock M 2003 Polymorphism in the collagen-like region of the *Bacillus anthracis* BclA protein leads to variation in exosporium filament length. J Bacteriol 185:1555–1563. doi:10.1128/JB.185.5.1555-1563.2003. 12591872 PMC148075

[19] Sylvestre P, Couture-Tosi E, Mock M 2005 Contribution of ExsFA and ExsFB proteins to the localization of BclA on the spore surface and to the stability of the *bacillus anthracis* exosporium. J Bacteriol 187:5122–5128. doi:10.1128/JB.187.15.5122-5128.2005. 16030205 PMC1196022

[20] Ohye DF, Murrell WG 1973 Exosporium and spore coat formation in *Bacillus cereus* T. J Bacteriol 115:1179–1190. 4199508 10.1128/jb.115.3.1179-1190.1973 PMC246368

[21] Steichen CT, Kearney JF, Turnbough CL Jr. 2007 Non-uniform assembly of the *Bacillus anthracis* exosporium and a bottle cap model for spore germination and outgrowth. Mol Microbiol 64:359–367. doi:10.1111/j.1365-2958.2007.05658.x. 17493122

[22] Terry C, Jiang S, Radford DS, Qiang W, Tzokov S, Moir A, Bullough PA 2017 Molecular tiling on the surface of a bacterial spore- the exosporium of the Bacillus anthracis/cereus/thuringiensis group. Mol Microbiol 104:539–552. doi:10.1111/mmi.13650. 28214340 PMC5434927

[23] Thompson BM, Stewart GC 2008 Targeting of the BclA and BclB proteins to the *Bacillus anthracis* spore surface. Mol Microbiol 70:421–434. doi:10.1111/j.1365-2958.2008.06420.x. 18761690

[24] Thompson BM, Waller LN, Fox KF, Fox A, Stewart GC 2007 The BclB glycoprotein of *Bacillus anthracis* is involved in exosporium integrity. J Bacteriol 189:6704–6713. doi:10.1128/JB.00762-07. 17644587 PMC2045162

[25] Boydston JA, Chen P, Steichen CT, Turnbough CL Jr. 2005 Orientation within the exosporium and structural stability of the collagen-like glycoprotein BclA of *Bacillus anthracis*. J Bacteriol 187:5310–5317. doi:10.1128/JB.187.15.5310-5317.2005. 16030225 PMC1196033

[26] Steichen C, Chen P, Kearney JF, Turnbough CL Jr. 2003 Identification of the immunodominant protein and other proteins of the *Bacillus anthracis* exosporium. J Bacteriol 185:1903–1910. doi:10.1128/JB.185.6.1903-1910.2003. 12618454 PMC150129

[27] Steichen CT, Kearney JF, Turnbough CL Jr. 2005 Characterization of the exosporium basal layer protein BxpB of *Bacillus anthracis*. J Bacteriol 187:5868–5876. doi:10.1128/JB.187.17.5868-5876.2005. 16109927 PMC1196169

[28] Thompson BM, Hsieh HY, Spreng KA, Stewart GC 2011 The co-dependence of BxpB/ExsFA and BclA for proper incorporation into the exosporium of *Bacillus anthracis*. Mol Microbiol 79:799–813. doi:10.1111/j.1365-2958.2010.07488.x. 21255119 PMC3044595

[29] Omotade TO, Bernhards RC, Klimko CP, Matthews ME, Hill AJ, Hunter MS, Webster WM, Bozue JA, Welkos SL, Cote CK 2014 The impact of inducing germination of *Bacillus anthracis* and *Bacillus thuringiensis* spores on potential secondary decontamination strategies. J Appl Microbiol 117:1614–1633. doi:10.1111/jam.12644. 25196092

[30] Omotade TO, Heffron JD, Klimko CP, Marchand CL, Miller LL, Halasahoris SA, Bozue JA, Welkos SL, Cote CK 2013 d-Cycloserine or similar physiochemical compounds may be uniquely suited for use in *Bacillus anthracis* spore decontamination strategies. J Appl Microbiol 115:1343–1356. doi:10.1111/jam.12322. 23927578

[31] Zheng LB, Donovan WP, Fitz-James PC, Losick R 1988 Gene encoding a morphogenic protein required in the assembly of the outer coat of the *Bacillus subtilis* endospore. Genes Dev 2:1047–1054. doi:10.1101/gad.2.8.1047. 3139490

[32] McPherson DC, Kim H, Hahn M, Wang R, Grabowski P, Eichenberger P, Driks A 2005 Characterization of the *Bacillus subtilis* spore morphogenetic coat protein CotO. J Bacteriol 187:8278–8290. doi:10.1128/JB.187.24.8278-8290.2005. 16321932 PMC1317010

[33] Driks A, Roels S, Beall B, Moran CP Jr, Losick R 1994 Subcellular localization of proteins involved in the assembly of the spore coat of *Bacillus subtilis*. Genes Dev 8:234–244. doi:10.1101/gad.8.2.234. 8299942

[34] Wang KH, Isidro AL, Domingues L, Eskandarian HA, McKenney PT, Drew K, Grabowski P, Chua MH, Barry SN, Guan M, Bonneau R, Henriques AO, Eichenberger P 2009 The coat morphogenetic protein SpoVID is necessary for spore encasement in *Bacillus subtilis*. Mol Microbiol 74:634–649. doi:10.1111/j.1365-2958.2009.06886.x. 19775244 PMC2806667

[35] Stevens C, Daniel R, Illing N, Errington J 1992 Characterization of a sporulation gene, spoIVA, involved in spore coat morphogenesis in *Bacillus subtilis*. J Bacteriol 174:586–594. doi:10.1128/jb.174.2.586-594.1992. 1729247 PMC205753

[36] Roels S, Driks A, Losick R 1992 Characterization of spoIVA, a sporulation gene involved in coat morphogenesis in *Bacillus subtilis*. J Bacteriol 174:575–585. doi:10.1128/jb.174.2.575-585.1992. 1729246 PMC205752

[37] McKenney PT, Driks A, Eskandarian HA, Grabowski P, Guberman J, Wang KH, Gitai Z, Eichenberger P 2010 A distance-weighted interaction map reveals a previously uncharacterized layer of the *Bacillus subtilis* spore coat. Curr Biol 20:934–938. doi:10.1016/j.cub.2010.03.060. 20451384 PMC2920530

[38] McKenney PT, Driks A, Eichenberger P 2013 The *Bacillus subtilis* endospore: assembly and functions of the multilayered coat. Nat Rev Microbiol 11:33–44. doi:10.1038/nrmicro2921. 23202530 PMC9910062

[39] Bailey-Smith K, Todd SJ, Southworth TW, Proctor J, Moir A 2005 The ExsA protein of *Bacillus cereus* is required for assembly of coat and exosporium onto the spore surface. J Bacteriol 187:3800–3806. doi:10.1128/JB.187.11.3800-3806.2005. 15901704 PMC1112046

[40] Takamatsu H, Kodama T, Nakayama T, Watabe K 1999 Characterization of the *yrbA* gene of *Bacillus subtilis*, involved in resistance and germination of spores. J Bacteriol 181:4986–4994. 10438771 10.1128/jb.181.16.4986-4994.1999 PMC93988

[41] Ozin AJ, Samford CS, Henriques AO, Moran CPJ 2001 SpoVID guides SafA to the spore coat in *Bacillus subtilis*. J Bacteriol 183:3041–3049. doi:10.1128/JB.183.10.3041-3049.2001. 11325931 PMC95203

[42] Ozin AJ, Henriques AO, Yi H, Moran CPJ 2000 Morphogenetic proteins SpoVID and SafA form a complex during assembly of the *Bacillus subtilis* spore coat. J Bacteriol 182:1828–1833. doi:10.1128/JB.182.7.1828-1833.2000. 10714986 PMC101864

[43] Qin H, Driks A 2013 Contrasting evolutionary patterns of spore coat proteins in two Bacillus species groups are linked to a difference in cellular structure. BMC Evol Biol 13:261–273. doi:10.1186/1471-2148-13-261. 24283940 PMC4219348

[44] Sambrook J, Fritsch EF, Maniatis T (ed). 1989 Molecular cloning: a laboratory manual. Cold Spring Harbor Laboratory Press, Cold Spring Harbor, NY.

[45] Koehler TM, Dai Z, Kaufman-Yarbray M 1994 Regulation of the *Bacillus anthracis* protective antigen gene: CO_2_ and a *trans*-acting element activate transcription from one of two promoters. J Bacteriol 176:586–595. doi:10.1128/jb.176.3.586-595.1994. 8300513 PMC205094

[46] Janes BK, Stibitz S 2006 Routine markerless gene replacement in *Bacillus anthracis*. Infect Immun 74:1949–1953. doi:10.1128/IAI.74.3.1949-1953.2006. 16495572 PMC1418658

[47] Plaut RD, Stibitz S 2015 Improvements to a markerless allelic exchange system for *Bacillus anthracis*. PLoS One 10:e0142758. doi:10.1371/journal.pone.0142758. 26624016 PMC4666636

[48] Sullivan MA, Yasbin RE, Young FE 1984 New shuttle vectors for *Bacillus subtilis* and *Escherichia coli* which allow rapid detection of inserted fragments. Gene 29:21–26. doi:10.1016/0378-1119(84)90161-6. 6092222

[49] Thompson BM, Hoelscher BC, Driks A, Stewart GC 2012 Assembly of the BclB glycoprotein into the exosporium and evidence for its role in the formation of the exosporium ‘cap’ structure in *Bacillus anthracis*. Mol Microbiol 86:1073–1084. doi:10.1111/mmi.12042. 22989026 PMC3508365

[50] Zorzoli A, Grayczyk JP, Alonzo F 2016 *Staphylococcus aureus* tissue infection during sepsis is supported by differential use of bacterial or host-derived lipoic acid. PLoS Pathog 12:e1005933. doi:10.1371/journal.ppat.1005933. 27701474 PMC5049849

[51] Boone TJ, Tyrrell GJ 2012 Identification of the actin and plasminogen binding regions of group B streptococcal phosphoglycerate kinase. J Biol Chem 287:29035–29044. doi:10.1074/jbc.M112.361261. 22761440 PMC3436549

