# Supplementary figures and images for: Coordinated Assembly of the Bacillus anthracis Coat and Exosporium during Bacterial Spore Outer Layer Formation
Source: mBio. 2018 Nov 6;9(6):e01166-18. doi: 10.1128/mBio.01166-18 (PMC6222130; doi:10.1128/mBio.01166-18)

15% SDS-PAGE

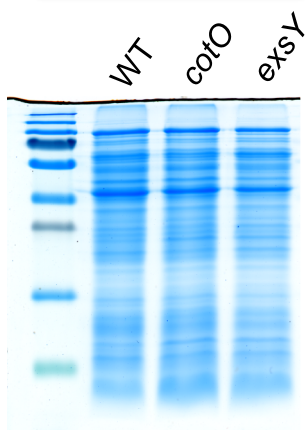

10% SDS-PAGE

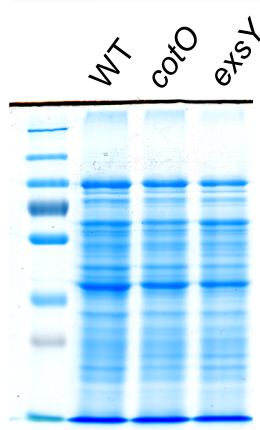

15% SDS-PAGE of sporangial proteins

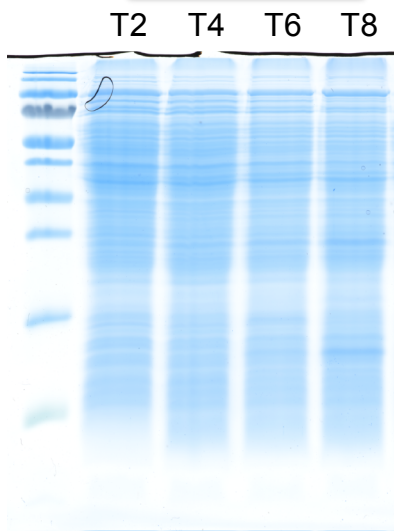

Supplement: FIG S1 [file mbo005184143sf1.pdf]

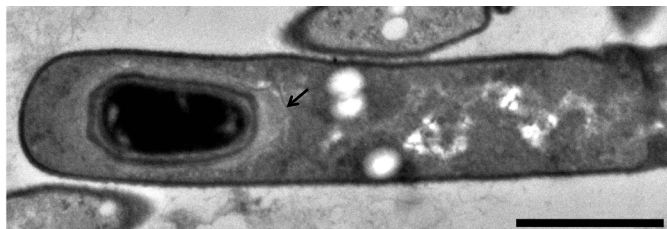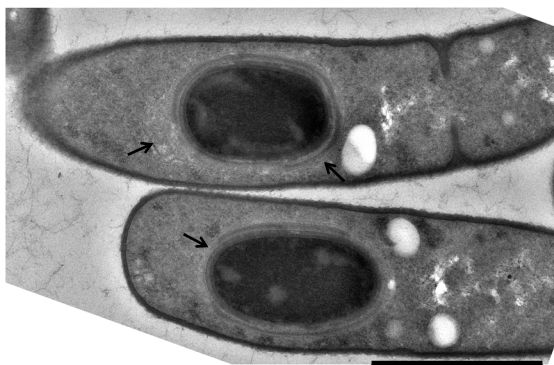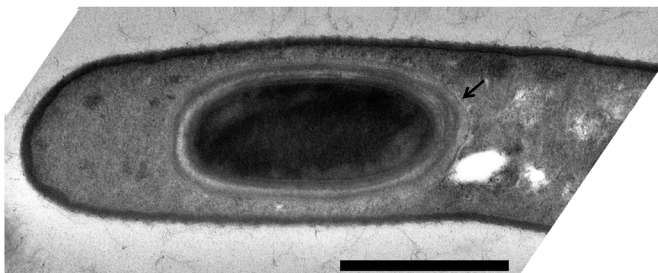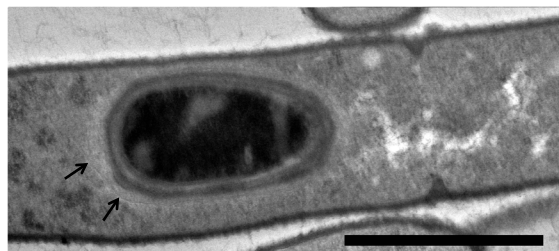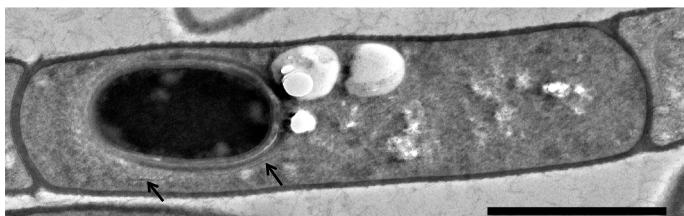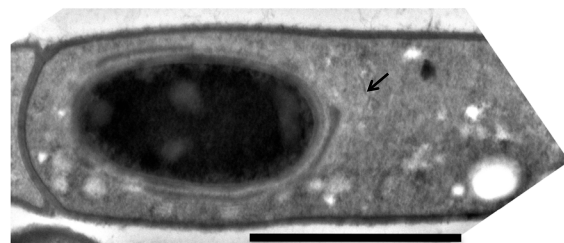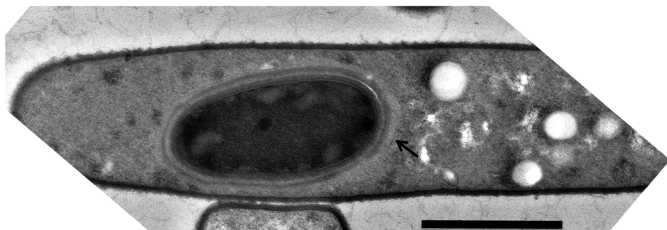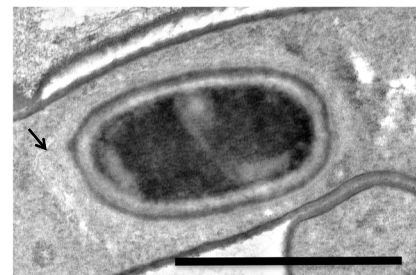

Supplement: FIG S3 [file mbo005184143sf3.pdf]
