# Supplementary material for: Coordinated Assembly of the Bacillus anthracis Coat and Exosporium during Bacterial Spore Outer Layer Formation
Source: mBio. 2018 Nov 6;9(6):e01166-18. doi: 10.1128/mBio.01166-18 (PMC6222130; doi:10.1128/mBio.01166-18)
Supplement: TABLE S1 [file mbo005184143st1.docx]

| **Primer Name** | **Sequence** |
| --- | --- |
| CotOnull-kpnF | GGTACCTGGGAGGGACGAGTGTGAAGAATA |
| CotOnull-hinR | AAGCTTGGCGGCATAATGGCTACAATTCCA |
| CotOup-sacF | TTAGAGCTCAGTGCATTCTCTTTAACAGC |
| CotOup-salR2 | CCGGTCGACCGTAGCTGAATAAATCGTG |
| CotOdown-salF | CCGGTCGACGGTGGTAGAAACCTTCTACC |
| CotOdown-kpnR | AATGGTACCGACCAAATCTCCTCAACTCC |
| cotYup-sacF | TTAGAGCTCACCTTTTCAAGTGAAAAAGG |
| cotYup-salR | CCGGTCGACTGTTCACTCCTTCTCGTGAG |
| cotYdwn-salF3 | CCGGTCGACTCAATGCTTGCGTGATGTTACG |
| cotYdwn-kpnR3 | AATGGTACCTGCAACTGTTAATGCTGCTG |
| ExsYup-kpnF | AATGGTACCCTCCGCTTTTTCTCGTATTATG |
| ExsYup-salR | CCGGTCGACGTCACTCCTTATCTTCTTGTTTG |
| ExsYdown-salF | CCGGTCGACCGTTACTATCTAATAAGTGAAG |
| ExsYdown-sacR | TTAGAGCTCGTAAGTTTGGAACTTGCTGC |
| pRP1028seqF | GTCGGGAATTGATGCAGAAGC |
| pRP1028seqR | CGGTACTATAGGGATAACAGG |
| EA1-pstIF | ATACTGCAGGCATTAAATCTTCAAACCACG |
| EA1-kpnR | TCGGGTACCTTATAGATTTGGGTTATTAAG |
| EA1-salF | ATTGTCGACGCATTAAATCTTCAAACCACG |
| CotO-SacF | TTAGAGCTCAGGACAATCTCTACATGTTG |
| CotO-salR | ATTGTCGACTTAAAAGCCGGCCATATCAATC |
| CotO-bamF | TTTGGATCCAGGACAATCTCTACATGTTG |
| CotO-pstR | CGGCTGCAGTTAAAAGCCGGCCATATCAATC |
| cotOproSm | CCCGGGGCACGCGGCGTAACAGGAGG |
| cotO3porfNh | GCTAGCAAAGCCGGCCATATCAATCG |
| cotE-bamF | CTTGGATCCTCCGAATTTAGAGAGATTATTAC |
| cotE-pstR | TATGGATCCTTACTCTTCTTCTGCATCAACG |
| pQEseqF | CCCGAAAAGTGCCACCTG |
| pQEseqR | GTTCTGAGGTCATTACTGG |

| **Plasmids** | **Description** | **Reference** |
| --- | --- | --- |
| pKS1 | kan^R^, erm^R^, repA | Shatalin + Neyfakh, 2005 |
| pMK4 | cm^R^, amp^R^, | Bacillus Genetic Stock Center |
| pDG4099 | pMK4 bearing the eGFP reporter | This Study |
| pBT4653 | pDG4099 expressing the cotO-gfp fusion | This Study |
| pRP1028 | spect^R^, Rfp, | Plaut, 2015 |
| pSS4332 | kan^R^, I-scEI | Plaut, 2015 |
| pSS1827 | amp^R^ | Plaut, 2015 |
| pMGM3 | pKS1 bearing an internal fragment of cotO | This study |
| pTJB57 | pMK4 containing the cotO under its native promoter | This study |
| pTJB104 | pRP1028 bearing 5' and 3' flanking regions of exsY | This study |
| pTJB126 | pTJB186 containing cotO under its native promoter | This study |
| pTJB128 | pRP1028 bearing 5' and 3' flanking regions of cotO | This study |
| pTJB186 | pRP1028 containing an internal 3' fragment of EA1 | This study |
| pTJB238 | pRP1028 5' and 3' flanking regions of cotY | This study |
| pQE30 | amp^R^, lacO, colE1 | Boone 2011 |
| pTJB81 | pQE30 containing the cotE gene | This study |
| pTJB166 | pQE30 containing the cotO gene | This Study |

| **Bacterial strains** | **Genotype** | **Source** |
| --- | --- | --- |
| ***E. coli*** |  |  |
| Subcloning efficiency DH5α | F- Φ80*lac*ZΔM15 Δ(*lac*ZYA-*arg*F) U169 *rec*A1 *end*A1 | Invitrogen |
|  | hsdR17(rk-, mk+) phoA supE44thi-1 gyrA96 relA1 λ- |  |
| One shot Top10 | F- *mcrA* Δ( *mrr-hsd*RMS-*mcr*BC) Φ80*lac*ZΔM15 Δ *lac*X74 | Invitrogen |
|  | recA1 araD139 Δ(araleu)7697 galU galK rpsL (StrR) endA1 nupG |  |
| ADL453 | JM109 *dam*::cm | Driks Laboratory Collection |
| TJB 25 | JM101 [pQE30] | Boone 2011 |
| TJB 30 | M15 [pREP4] | Boone 2011 |
| TJB81 | M15 [pREP4] [pQE30-cotE] | This study |
| TJB166 | M15 [pREP4] [pQE30-cotO] | This study |
| ***B. anthracis* Sterne strain derivatives** |  |  |
| TJB3 | Wildtype 34F2 | Giorno 2007 |
| MGM76 | Sterne 34F2 cotOΩpMGM3 | This study |
| RG56 | Sterne cotEΔ::km | Giorno 2007 |
| TJB67 | MGM76 [pTJB 57] | This study |
| TJB116 | Sterne [pTJB57] | This study |
| TJB124 | Sterne 34F2 ΔcotO | This study |
| TJB130 | TJB124 eagΩpTJB11 | This study |
| TJB139 | Sterne 34F2 ΔexsY | This study |
| TJB239 | Sterne 34F2 ΔcotY | This study |
| MUS 8188 | Sterne [pBT4653] | This study |
| MUS 8189 | RG56 [pBT4653] | This study |
